# Thrombocytopenia in *Plasmodium vivax* Malaria Is Related to Platelets Phagocytosis

**DOI:** 10.1371/journal.pone.0063410

**Published:** 2013-05-28

**Authors:** Helena Cristina C. Coelho, Stefanie C. P. Lopes, João Paulo D. Pimentel, Paulo A. Nogueira, Fábio T. M. Costa, André M. Siqueira, Gisely C. Melo, Wuelton M. Monteiro, Adriana Malheiro, Marcus V. G. Lacerda

**Affiliations:** 1 Universidade do Estado do Amazonas, Manaus, Amazonas, Brazil; 2 Fundação de Medicina Tropical Dr. Heitor Vieira Dourado, Manaus, Amazonas, Brazil; 3 Universidade Estadual de Campinas, Campinas, São Paulo, Brazil; 4 Instituto Leônidas e Maria Deane, Fiocruz, Manaus, Amazonas, Brazil; 5 Universidade Federal do Amazonas, Manaus, Amazonas, Brazil; 6 Fundação de Hematologia e Hemoterapia do Amazonas, Manaus, Amazonas, Brazil; Centro de Pesquisa Rene Rachou/Fundação Oswaldo Cruz (Fiocruz-Minas), Brazil

## Abstract

**Background:**

Although thrombocytopenia is a hematological disorder commonly reported in malarial patients, its mechanisms are still poorly understood, with only a few studies focusing on the role of platelets phagocytosis.

**Methods and Findings:**

Thirty-five malaria vivax patients and eight healthy volunteers (HV) were enrolled in the study. Among vivax malaria patients, thrombocytopenia (<150,000 platelets/µL) was found in 62.9% (22/35). Mean platelet volume (MPV) was higher in thrombocytopenic patients as compared to non- thrombocytopenic patients (p = 0.017) and a negative correlation was found between platelet count and MPV (r = −0.483; p = 0.003). Platelets from HV or patients were labeled with 5-chloromethyl fluorescein diacetate (CMFDA), incubated with human monocytic cell line (THP-1) and platelet phagocytosis index was analyzed by flow cytometry. The phagocytosis index was higher in thrombocytopenic patients compared to non-thrombocytopenic patients (p = 0.042) and HV (p = 0.048). A negative correlation was observed between platelet count and phagocytosis index (r = −0.402; p = 0.016). Platelet activation was assessed measuring the expression of P-selectin (CD62-P) in platelets’ surface by flow cytometry. No significant difference was found in the expression of P-selectin between thrombocytopenic patients and HV (p = 0.092). After evaluating the cytokine profile (IL-2, IL-4, IL-6, IL-10, TNF-α, IFN-γ and IL-17) in the patients’ sera, levels of IL-6, IL-10 and IFN-γ were elevated in malaria patients compared to HV. Moreover, IL-6 and IL-10 values were higher in thrombocytopenic patients than non-thrombocytopenic ones (p = 0.044 and p = 0.017, respectively. In contrast, TNF-α levels were not different between the three groups, but a positive correlation was found between TNF-α and phagocytosis index (r = −0.305; p = 0.037).

**Conclusion/Significance:**

Collectively, our findings indicate that platelet phagocytosis may contribute to thrombocytopenia found in vivax malaria. Finally, we believe that this study opens new avenues to explore the mechanisms involved in platelet dysfunction, commonly found in vivax malaria patients.

## Introduction

*Plasmodium* infections are still a major public health problem, resulting in millions of deaths annually worldwide [1]. Although *P. falciparum* is responsible for the majority of severe complications cases and malaria-associated mortality [2]; vivax malaria has now clearly emerged as a potentially lethal condition [3], [4], despite of having previously been considered a benign disease. *P. vivax* is more widely distributed than *P. falciparum* and has potential to cause morbidity and mortality amongst the 2.85 billion people living at risk of infection [5]. In Brazil, *P. vivax* accounts for up to 80% of the malaria cases [6].

Thrombocytopenia and anemia are the most common malaria-associated hematological complications in *P. vivax* and *P. falciparum* [7]. High frequency of thrombocytopenia in patients with malaria has been well-documented in several studies [8], including reports from Manaus in the Brazilian Amazon [8], [9]. Indeed, Kochar and colleagues have recently shown that severe thrombocytopenia (platelet count <20×10^3^/mm^3^) is a common manifestation in patients with vivax mono-infection confirmed by PCR [10], [11].

Research on the pathogenesis of malaria thrombocytopenia has been conducted for more than four decades, however the exact mechanism underlying this phenomenon remains not elucidated. Nevertheless, thrombocytopenia in malaria seems to be a multifactorial phenomenon and probably involves an increase in platelets destruction and consumption [12]. Moreover, although some studies showed bleeding associated with thrombocytopenia in malaria [11], [13], low platelet counts were not commonly accompanied by severe bleeding [8].

Several mechanisms have been proposed to explain malaria thrombocytopenia [12], [14]–[20]. Some studies suggest that the low platelet counts in malaria might be caused by activation [20] and/or apoptosis of platelets [14], thus leading to its removal by the immune system [12], [15]. Nonetheless, it has also been proposed that immune complexes generated by malarial antigen could lead to sequestration of the injured platelets in the spleen followed by phagocytosis by splenic macrophages [16]–[19].

Recently, Klein and Ronez [21] showed a blood smear from a *P. falciparum* patient compatible with peripheral hemophagocytosis. This patient presented marked thrombocytopenia and platelet-like particle inside the monocytes [21]. Indeed, platelet phagocytosis in malaria was shown more than 20 years ago in a patient report with 80% of circulating monocytes presenting platelets inside [22].

Although there are some evidences of phagocytosis involvement in malaria thrombocytopenia, information regarding the mechanisms responsible for this phenomenon is scarce. Herein, we investigate the role of platelets phagocytosis in malaria vivax thrombocytopenia, after establishing an *in vitro* phagocytosis assay based on flow cytometry in the presence of platelets from patients and healthy donors and THP-1 cells.

## Materials and Methods

### Ethics Statement

All protocols and consents forms were approved by the Ethics Review Board of the *Fundação de Medicina Tropical Dr. Heitor Vieira Dourado* (*FMT-HVD*) (approval number 1610–11). A signed informed consent was obtained from each subject enrolled in this study.

### Study Area and Subjects

Patients were recruited and examined at FMT-HVD, a tertiary care center for infectious diseases in Manaus, the capital of the Amazonas State, Brazil. Up to 20 mL of peripheral blood was collected immediately after confirmation of *P. vivax* infection by thick blood smear (n = 35). Afterwards, patients were treated with chloroquine and primaquine, according to the standard protocol recommended by the *Brazilian Malaria Control Program*. *P. vivax* mono-infection was subsequently confirmed by polymerase chain reaction (PCR) analysis [23]. Peripheral blood was also collected from eight healthy volunteers (HV) living in Manaus, negative for *P. vivax* infection by thick blood film and PCR and with no previous history of malaria.

Clinical and demographical data were acquired through a standardized questionnaire, and the hematological profile, including peripheral platelet count and MPV, were determined using a cell counter (Sysmex KX-21N®). Patients presenting any other co-morbidity related to thrombocytopenia that could be traced were excluded from the study, as well as HVs. The co-morbidities investigated were human immunodeficiency virus (HIV) (Rapid Check HIV 1&2®), dengue (Dengue Eden Test Bioeasy®, MG, Brazil), leptospirosis (SD Bioline Leptospira IgG/IgM, Kyonggi-do®, Korea), hepatitis C (Anti-HCV Bioeasy®, MG, Brazil) and hepatitis B (HBsAg ELISA Bioeasy®, MG, Brazil).

### Platelets Isolation

Platelets were isolated from whole blood collected in sodium citrate solution (3.8%) from vivax malaria patients or HVs and centrifuged for 10 min at 200×g to generate platelet-rich plasma (PRP). To avoid platelets aggregation and activation, PRP was acidified with citric acid 0,15 M until the PRP pH reached 6.4 and then 1 µg/mL of prostaglandin E-1 (PGE-1) was added to avoid platelet stimulation. PRP was pelleted by centrifugation for 10 min at 1,600×g and the platelets pellet was re-suspended in phosphate buffered saline (PBS) supplemented with 0.5% bovine serum albumin, 2mM EDTA, 0.1% sodium azide and 1 µg/mL PGE-1. For phagocytosis experiments, platelets were fluorescently labeled with 5 µg of CellTracker® Green CMFDA (Invitrogen®) by 60 min incubation at 37°C, followed by two washes in supplemented PBS. The number of platelets was determined and the solution was adjusted to 50×10^6^ platelets/mL. The efficiency of platelet labeling with CMFDA was determined to be above 95% using flow cytometry (FACS Calibur®, BD Biosciences®, San Jose, CA).

### P-selectin Expression

P-selectin expression in platelets (chosen as a surrogate of platelet activation) was measured in two moments, in the PRP and after platelet isolation. For this purpose 100 µL of PRP or platelet solution (5×10^6^ platelets/mL) were incubated with 4 µL of PE mouse anti-human CD62-P (BD Pharmingen®) for 30 min at 37°C. After two washes in supplemented PBS, P-selectin expression was measured on a FACScalibur® (BD Biosciences®, San Jose, CA).

### THP-1 Cells

Human monocytic THP-1 cells (ATCC® TIB-20®) were cultured in RPMI-1640 medium (Gibco®) supplemented with 10% fetal calf serum (FCS) and gentamicin (40 µg/L) at 37°C. THP-1 cells were counted in a Neubauer chamber and 1×10^6^ cells per well were added in a 24 wells plate. Maturation was induced by incubation with 60 ηg/mL of Phorbol 12-Myristate 13-Acetate (PMA) (Calbiochem®, San Diego, CA) for 2 hours at 37°C. After this period, the supernatant was removed and the THP-1 cells were washed twice with RPMI medium.

### In vitro Platelet Phagocytosis

After cell maturation, phagocytosis of platelets by THP-1 cells was measured by flow cytometry as previously described [24]. Briefly, 5×10^6^ fluorescently labeled platelets were added to each well and then plates were centrifuged at 500×g for 5 min at room temperature. After 60 min of incubation in 5% CO_2_ atmosphere at 37°C, the THP-1 cells were harvested, washed three times in PBS and fixed in paraformaldehyde 4% in cacodylate buffer for flow cytometry analysis.

### Flow Cytometry Analysis

The THP-1 cells were gated and 10,000 events were acquired from each sample. The frequency of platelet phagocytosis (FP) was determined by counting the CellTracker® Green CMFDA positive cells in FL1-H. The median intensity of fluorescence (MIF) emitted for each cell was also evaluated. As larger platelets have a greater amount of CellTracker® Green CMFDA, the mean platelet volume (MPV) may affect the intensity of fluorescence. Then, to standardize the platelet phagocytosis for each sample, we created a formula to calculate the Phagocytosis index: PI = MIF×FP/100×MPV.

### Cytokine Measurements

The levels of IL-2, IL-4, IL-6, IL-10, IL-17, IFN-γ and TNF-α were quantified in cryopreserved serum using the Cytometric Bead Array kit (CBA, BD Biosciences Pharmingen®) following manufacturer’s instructions. All the cytokine levels below detection limit were given half of the threshold value and those values above the upper detection limit were excluded from the analysis.

### Statistical Analysis

All data were expressed as the mean ± SD. Correlations were analyzed using the Spearman correlation. Normal distribution of data was evaluated with the Kolmogorov-Smirnov test. Comparisons between groups were analyzed using the Mann-Whitney U test (two groups) or Kruskal Wallis test. P-selectin expression before and after platelets isolation were compared by Wilcoxon signed rank test. Differences were considered statistically significant when p≤0.05. Statistical analysis was performed using the GraphPad Prism® version 5.0 (GraphPad Software®, CA, US).

## Results

### Patient’s Characteristics and Thrombocytopenia Frequency

According to Table 1, thrombocytopenia (<150,000 platelets/µL) was found in 62.9% of the patients (22/35) enrolled in this study. Amongst thrombocytopenic patients, 18.2% (4/22) presented severe thrombocytopenia (<50,000 platelets/µL). Moreover, no significant difference in duration of clinical malaria symptoms and number of previous malaria episodes were observed between thrombocytopenic and non-thrombocytopenic patients (Table 1). Likewise, the frequencies of primary infection and past malaria infection in the last six months were similar in both groups (Table 1).

**Table 1:** Characteristics of patients with *Plasmodium vivax* (with and without thrombocytopenia).

Characteristics		Patients			P value*
		Total	NT	T	
Age, years (mean ±SD)		41.8±13.6	40.1±13.8	42.4±13.9	0.918^a^
Sex (%)	*M*	29/35 (82.9)	9/13(69.3)	19/22 (86.4)	0.383^b^
	*F*	6/35 (17.1)	4/13(30.7)	3/22 (13.6)	
Duration of symptoms in days (mean ±SD)		5.5±4.0	5.3±3.5	5.3±4.9	0.605^a^
Previous malaria episodes (%)	*Yes*	27/35 (77.2)	11/13 (84.6)	16/22 (72.7)	0.680^b^
	*No*	8/35 (22.8)	2/13 (15.4)	6/22 (27.3)	
N° of previous malaria episodes (mean ±SD)		3.6±3.6	3.1±2.9	3.9±4.0	0.876^a^
Last malaria (%)	*<6 months*	13/27 (48.2)	7/16 (43.7)	6/11 (54.6)	0.581^b^
	*≥6 months*	14/27 (51.8)	9/16 (56.3)	5/11 (45.4)	

SD = standard deviation.

NT = non-thrombocytopenic; T = thrombocytopenic.

* Non-thrombocytopenic patients×thrombocytopenic patients.

a Mann Whitney test.

b Chi-square or Fisher’s exact test.

### MPV and Thrombocytopenia

**Figure 1:**
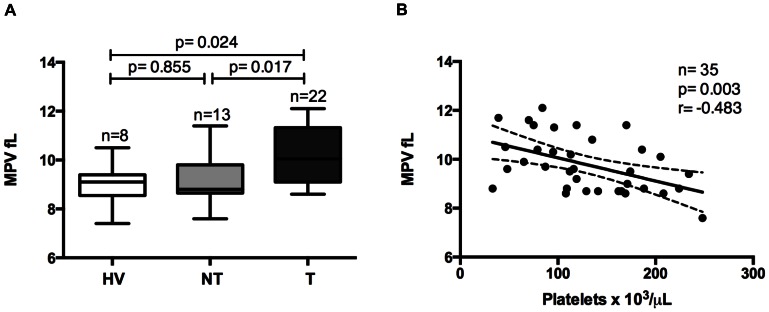
Mean platelet volume (MPV). MPV comparisons between healthy volunteers (HV), non-thrombocytopenic (NT) and thrombocytopenic (T) patients with vivax malaria (A).

MPV was significantly elevated in patients with thrombocytopenia (Figure 1A). Moreover, a negative correlation was observed between the MPV and the platelet count in malaria patients (r = −0.483; p = 0.003) (Figure 1B).

### Parasitemia and Thrombocytopenia

**Figure 2:**
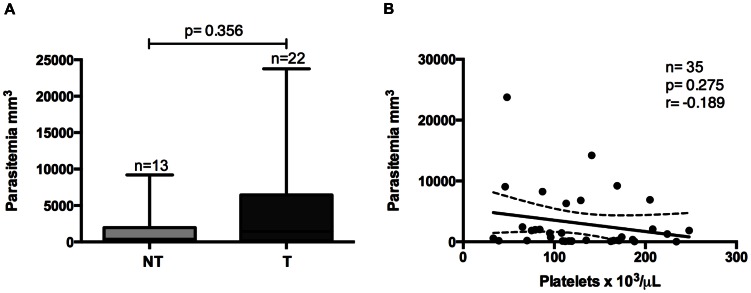
Parasitemia and thrombocytopenia. Comparisons of parasitemia (per mm^3^ of blood) between non-thrombocytopenic (NT) and thrombocytopenic (T) patients with malaria (A). Correlation between platelet count and parasitemia (mm^3^ of blood) (B).

Parasitemia was similar in thrombocytopenic and non-thrombocytopenic patients (Figure 2A) and no correlation was found between platelet count and parasitemia (Figure 2B).

### Phagocytosis Assay

**Figure 3:**
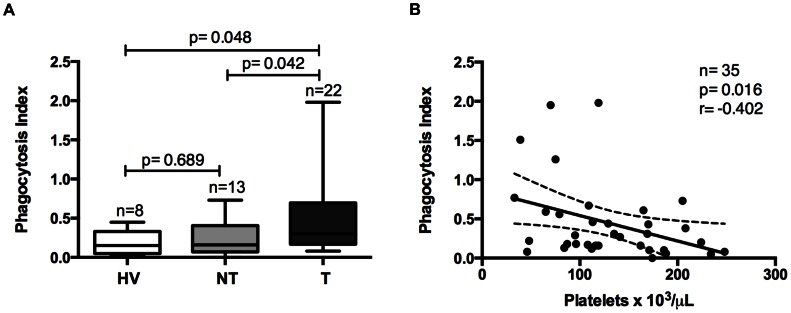
Phagocytosis index (PI). Comparisons of phagocytosis between healthy volunteers (HV), non-thrombocytopenic (NT) and thrombocytopenic (T) patients with malaria (A). Correlation between platelet count and phagocytosis index (B).

The phagocytosis index was significantly higher in patients with thrombocytopenia malaria than in patients without thrombocytopenia (p = 0.042) and HV (p = 0.048) (Figure 3A). Moreover, significantly correlation was observed between platelet count and phagocytosis index (r = −0.426; p = 0.016) (Figure 3B). Phagocytosis index not corrected by MPV was also analyzed and lead to the same results (data not shown).

### P-selectin Expression

**Figure 4:**
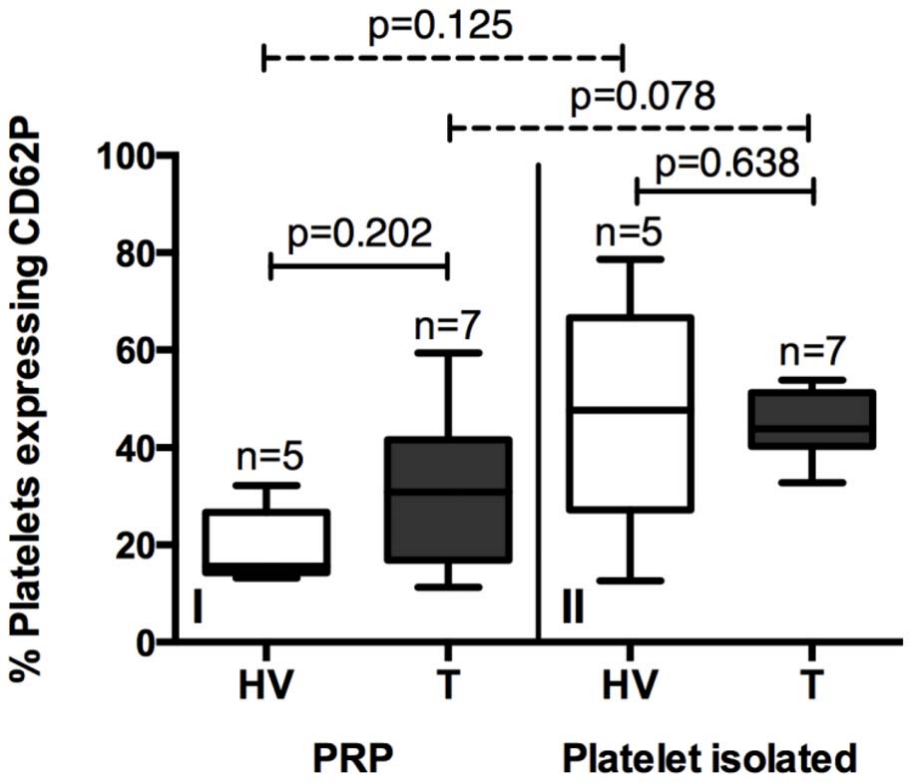
P-selectin expression in healthy volunteers an thrombocytopenic patients. The P-selectin expression in platelets was measured in two moments, in the PRP and in isolated platelets.

P-selectin expression was similar between thrombocytopenic patients and HVs in two time-points: immediately after harvesting (PRP) or after washing and CMFDA labeling. According to Figure 4, no significant increase in P-selectin expression was found in platelet isolation process for either non-thrombocytopenic or thrombocytopenic patients.

### Cytokine Profile in Patients’ Sera

**Figure 5:**
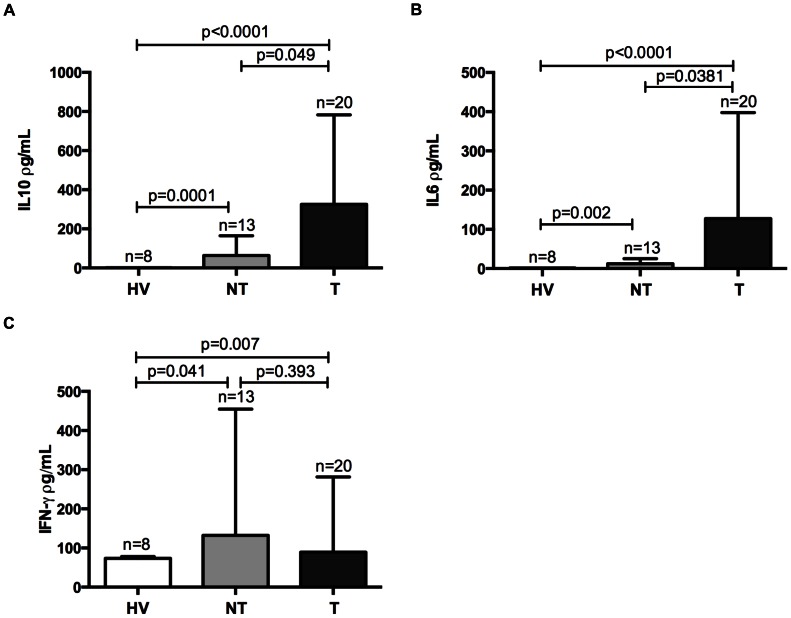
Cytokines levels. Comparisons of IL-6 (A), IL-10 (B), and IFN-γ (C) between healthy volunteers (HV), non-thrombocytopenic (NT) and thrombocytopenic (T) patients.

**Figure 6:**
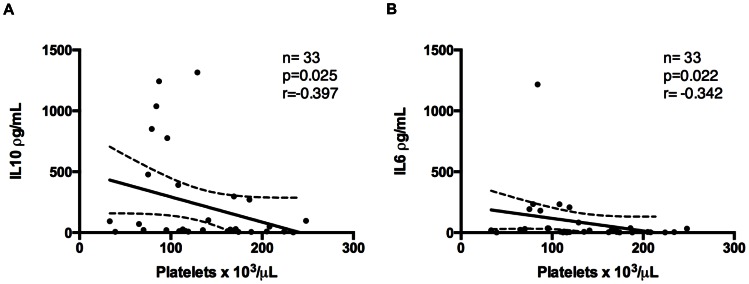
Correlation between cytokines levels and platelet count. Correlation between IL-6 (A) and IL-10 (B) and platelet count.

**Figure 7:**
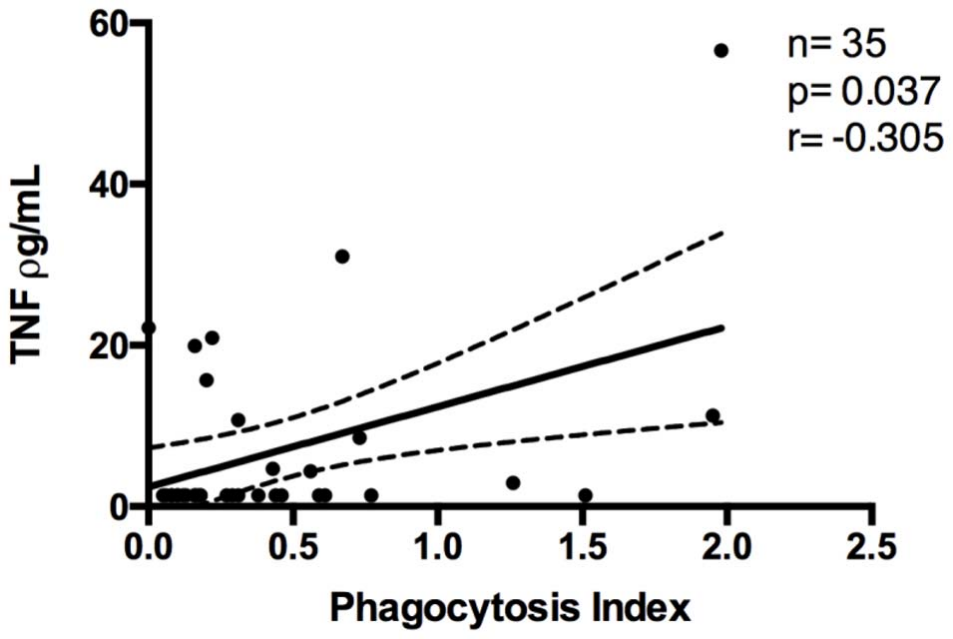
Correlation between TNF-α and phagocytosis index (PI).

Of seven cytokines analyzed in this study, IL-6, IL-10 and IFN-γ were elevated in malaria patients sera, thrombocytopenic or not, compared to HVs (Figure 5A, 5B and 5C). IL-6 and IL-10 were higher in thrombocytopenic patients than in non-thrombocytopenic ones (Figure 5A and 5B). Indeed, negative correlations were found between platelet counts and IL-6 and IL-10 values (Figure 6A and 6B). A positive correlation was found only between phagocytosis index and TNF-α values (Figure 7).

## Discussion

Despite not being a criterion for severe malaria, thrombocytopenia is one of the most common complications of both *P. vivax* and *P. falciparum* malaria. Recently, Kochar and colleagues have shown that thrombocytopenia is more frequent and severe among patients with *P. vivax* infection [10]. Nevertheless, only a limited number of studies have addressed key questions on the pathogenesis of thrombocytopenia in malaria. Of those, two independent studies have shown platelets phagocytosis in malaria thrombocytopenic patients [21], [22], although a detailed investigation of this phenomenon was not pursued. Herein, by means of an *in vitro* phagocytosis assay, we evaluated the involvement of platelet phagocytosis in vivax malaria thrombocytopenia.

In this study, thrombocytopenia was frequently detected amongst vivax malaria patients (62.9%) as well as severe thrombocytopenia (platelet counts under 50,000 platelets/µL) (18.2%). Nevertheless, we did not observe association between severe thrombocytopenia and bleeding in these patients, although severe thrombocytopenia is occasionally associated with severity [25], [26] including severe vivax patients [27], [28].

In this study, MPV was elevated in thrombocytopenic patients and a negative correlation between platelet counts and MPV was detected in malaria patients. Our findings corroborates previous studies [11] and are in line with the rationale that larger platelets observed in thrombocytopenic patients may be a manner to compensate the low absolute number of platelets in the periphery; therefore preserving primary hemostasis and avoiding severe bleeding [8].

Negative correlation between parasitemia and thrombocytopenia has been shown elsewhere [29]–[31], and this correlation has been attributed to platelets shortened lifespan due to immune complexes sequestration in their surface [16]–[18]. Surprisingly, we did not find any relation between parasitemia and platelet counts in vivax malaria patients. Despite of our small sample size, findings corroborate a large study conducted in Bikaner, India [32]. Indeed, despite the fact that circulating immune complexes are elevated in vivax and falciparum malaria, their role in the development of thrombocytopenia is not clear [33], [34]. Nonetheless, we observed a negative correlation between platelet counts and phagocytosis index, indicating that platelet phagocytosis may be involved in thrombocytopenia pathogenesis in vivax malaria.

It has been proposed that platelet phagocytosis could be mediated by the increase in P-selectin expression in the surface of activated platelets [35]. However, only two studies evaluated P-selectin expression in malaria thrombocytopenia [20], [36], and just one in *P. vivax* malaria [20]. Recently, de Mast and colleagues showed that P-selectin expression in platelets surface and circulating P-selectin in plasma were not associated with low platelet count in *P. falciparum* experimentally infected volunteers [36]. In contrast, Lee and colleagues showed that circulating P-selectin in plasma was elevated in *P. falciparum* severe malaria but not in *P. vivax* or *P. falciparum* non-severe infections [20]. As P-selectin expression levels were not augmented in the platelets from thrombocytopenic patients in our study, we believe that this molecule is not directly involved in platelet phagocytosis.

Cytokines released during an acute inflammatory response could contribute to the pathogenesis of thrombocytopenia. Recently, a study showed that the administration of IL-10 to healthy volunteers was capable of inducing thrombocytopenia [37]. This decrease in platelet counts in IL-10 treated group was accompanied by reduction in the amount of megakaryocyte colony-forming units, indicating the participation of this cytokine in platelet production [37]. Actually, it has been shown that thrombocytopenia in children with acute falciparum malaria is strongly associated with plasma concentrations of IL-10, but not with *P. falciparum* parasitemia or other plasma cytokines [38]. Park and colleagues showed higher levels of IL-1, IL-6, IL-10 and TGF-ß in *P. vivax* thrombocytopenic patients compared to non-thrombocytopenic [39]. Indeed, similar to previous findings [38], [39], we observed that IL-6 and IL-10 levels are elevated in thrombocytopenic patients serum compared to non-thrombocytopenic ones, and negative correlations between IL-6 and IL-10 levels and platelet count were found.

TNF-α has been associated with platelet consumption in mice but not with platelet production [40]. In our study, TNF-α levels were similar in malaria patients and HV but a positive correlation between TNF-α levels in serum and phagocytosis index was found. In contrast, IFN-γ was elevated in thrombocytopenic patients as compared to HV. In fact, high levels of IFN-γ and TNF-αare often correlated to severity in murine experimental models and in humans infected with *P. falciparum* and *P. vivax* [28], [41]–[44]. However, the relationship between thrombocytopenia and severe malaria is nebulous [8], [38], and further studies are needed to understand the pathogenesis associated with thrombocytopenia.

### Conclusion

Collectively, our findings demonstrate that platelet phagocytosis is associated to thrombocytopenia and correlates with TNF-α, a cytokine normally attributed to severity in malaria. Moreover, we showed that this increase in phagocytosis has not been associated with parasitemia or platelet activation. Importantly, our data brings new insights about the mechanisms involved in malaria vivax thrombocytopenia and highlights the potential relevance of this phenomenon.
